# The Associations of Vitamin D with Ovarian Reserve Markers and Depression: A Narrative Literature Review

**DOI:** 10.3390/nu16010096

**Published:** 2023-12-27

**Authors:** Gyun-Ho Jeon

**Affiliations:** Department of Obstetrics and Gynecology, Haeundae Paik Hospital, Inje University School of Medicine, Busan 48108, Republic of Korea; jeon285@hotmail.com; Tel.: +82-51-797-2020

**Keywords:** vitamin D, ovarian reserve, anti-Müllerian hormone, depression, reproduction, central nervous system

## Abstract

Since the identification of vitamin D receptors in both the female reproductive tract and the central nervous system, further data have shown that vitamin D is involved in the processes of reproductive and mental health. This paper reviews current research on the associations of vitamin D with ovarian reserve markers and depression and discusses the potential role of vitamin D in their relationships. There have been numerous studies reporting that vitamin D was significantly related to ovarian reserve markers and depression in basic or clinical research, but some observational and interventional clinical studies have shown inconsistent results. Nevertheless, recent meta-analyses of interventional studies have provided promising results showing that vitamin D supplementation significantly improves ovarian reserve metrics, especially in a subgroup of women with normal or diminished ovarian reserve, and decreases depressive symptoms and risk. The demonstration of an association of vitamin D with both ovarian reserve and depression could suggest that vitamin D may be another important key in explaining female reproductive depression. Larger-scale studies in standardized settings will be needed in order to gain further insight into the role of vitamin D in female reproduction and depression.

## 1. Introduction

Vitamin D was initially known to be essential for bone health and calcium phosphorus homeostasis [1], but recent research suggests that it may also have important roles in blood pressure regulation, glucose control, wound healing, and immune function, and is even associated with cancer, autoimmune disease, obesity, etc. [2,3,4,5]. Furthermore, vitamin D also may act on the regulation of the microbiome, the modulation of immune and inflammatory processes, and the release of antimicrobial peptides. Vitamin D deficiency has also been associated with many pathologies, including inflammatory bowel disease, colorectal cancer, and sclerosis [6]. More recently, research on the role of vitamin D in female reproductive health and depression has increased as vitamin D receptors (VDRs) have been detected throughout the female reproductive system and central nervous system (CNS) [7,8]. The biological actions of vitamin D are applied through a VDR, which is a ligand-dependent transcription factor located in the nucleus of target cells [9]. The vitamin D-nuclear receptor complex then acts as a transcription factor and exerts a genomic effect on the ovary or brain [8,10]. Although the exact genomic effects on the target cells and the underlying mechanism by which vitamin D may be involved in reproductive and CNS systems are unknown, a direct association between vitamin D and ovarian steroidogenesis or brain-derived neurotrophic factor (BDNF) levels from in vitro and in vivo studies has been suggested [11,12,13]. In this context, several studies have been published on the relationship between vitamin D and ovarian reserve markers, BDNF, and neurotrophic factors [14,15], and there have also been many clinical reports that vitamin D deficiency is associated with reproductive hormonal decline or imbalances such as menopause, polycystic ovarian syndrome (PCOS), primary ovarian insufficiency (POI), and depression in women [16,17,18,19]. Meanwhile, the relationship between female reproductive hormone changes and depression is already well established [20,21], and several studies have shown that depression in female reproductive hormone-related diseases is also associated with low vitamin D levels and ovarian reserve markers [22,23]. In this regard, vitamin D, which is involved in both female hormones and depression, may be another key to explaining female reproductive depression. This paper reviews current research on the association of vitamin D with ovarian reserve markers and depression and discusses the potential role of vitamin D in their relationships.

## 2. Materials and Methods

This article presents an overview of the association of vitamin D with ovarian reserve markers and depression. It also covers the possible relationship of vitamin D with certain ovarian-reserve-related reproductive depression. This work is based on an analysis of the literature from a thorough electronic search in PubMed (Medline), EMBASE, and Web of Science databases using the following keywords: ‘vitamin D’, ‘vitamin D deficiency’, ‘ovarian reserve’, ‘anti-Müllerian hormone (AMH)’, ‘depression’, ‘reproduction’, and ‘microbiota’, from their inceptions through July 2023. We included systematic reviews, meta-analyses, and review articles and referred to some single clinical studies that were not included in the meta-analyses if they were deemed relevant. To supplement our article base, we also added references from these selected articles and publications used in our previous work. We did not restrict our research to specific results but rather aimed to provide an up-to-date overview of all clinical data on the relation of vitamin D to ovarian reserve and depression. We also discuss the potential role of vitamin D in depression related to female reproductive hormones in a separate section.

## 3. The Association of Vitamin D and Ovarian Reserve Markers

Today, the most often used ovarian reserve marker is AMH, a gonadal-specific glycoprotein produced by granulosa cells of small antral or pre-antral follicles. AMH is also known to play a crucial role in folliculogenesis with minimal variations in its levels during the menstrual cycle, making it the most useful predictive marker for assisted reproductive technology [24]. Antral follicle counts (AFC) and basal follicular stimulation hormone (FSH) levels are also often used to represent ovarian reserve in clinical practice [25]. Research on the relationship between vitamin D and these markers representing ovarian reserves has been conducted in the form of cellular or genetic, clinical studies, and a few meta-analyses have been published recently. A summary of these studies is provided in Table 1.

### 3.1. Basic Studies at the Cellular and Genetic Level

**Figure 1:**
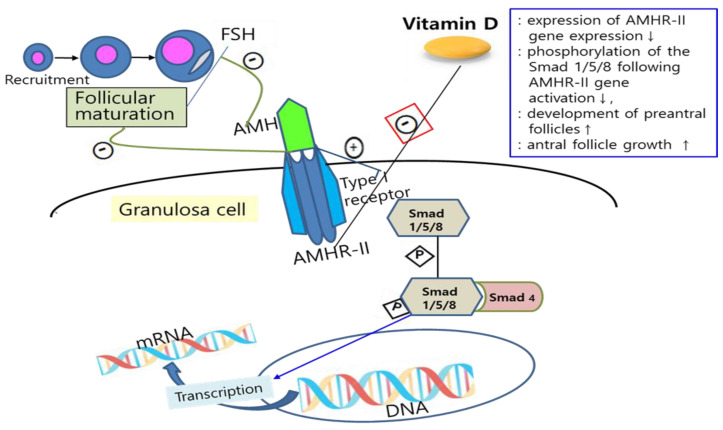
Mechanisms of the effects of vitamin D on AMH signaling and ovarian follicle development. Vitamin D alters the AMH signaling and actions by downregulation of AMHR-II gene expression (red box), inhibiting the phosphorylation of Smad and its nuclear localization, and can also promote follicle differentiation and development by altering AMH production patterns and FSH sensitivity in ovarian granulosa cells. FSH: follicle-stimulating hormone; AMH: anti-Müllerian hormone; AMHR-II: anti-Müllerian hormone receptor-II; mRNA: messenger ribonucleic acid; DNA: deoxyribonucleic acid; P: phosphate; ↑: increase; ↓: decrease.

There has been increased interest in the potential relationship between vitamin D and ovarian reserve markers, particularly AMH, which is important for folliculogenesis, as reports about the effects of vitamin D on follicular development and steroidogenesis in animal and human cell-line studies have shown. For example, VDR null mutant mice not only have impaired folliculogenesis but also show uterine hypoplasia, decreased aromatase activity, aromatase gene expression, and increased FSH levels [7,11]. Also, vitamin D stimulates steroidogenesis in human ovarian cells [12]. However, there is limited information about how vitamin D affects ovarian reserve markers such as AMH. Malloy et al. demonstrated the presence of functional vitamin D response elements in the human AMH gene promotor region and a direct effect of vitamin D on AMH expression via these response elements [26]. Wojtusik et al. observed a dose-dependent decrease in AMH mRNA levels in hen granulosa cells after treatment with vitamin D [27]. In another recent study, treatment of human granulosa cells with vitamin D exhibited altered AMH signaling and an inverse correlation between vitamin D status in follicular fluid and AMH receptor-II (AMHR-II) mRNA gene expression [10]. Binding AMH to AMHR-II activates the type I transmembrane receptor and, subsequently, phosphorylation of Smad 1/5/8, which interacts with Smad 4, followed by this complex regulating transcription of the target gene after moving into the nucleus [28,29]. Binding AMH to AMHR-II is also known to suppress follicular maturation by inhibiting primordial follicle recruitment into the growing follicle pool and by decreasing the sensitivity of follicles to FSH [30]. Since vitamin D plays a role in the downregulation of AMHR-II gene expression, inhibiting the phosphorylation of Smad and its nuclear localization, it is suggested that vitamin D may promote follicle differentiation and development by altering AMH production patterns and FSH sensitivity in ovarian granulosa cells [31,32] (Figure 1). Indeed, Xu et al. showed that vitamin D sustained AMH production and enhanced the antral follicle growth in a rhesus monkey ovarian cell line study [33]. However, there was a discrepancy between these in vitro studies and clinical studies, which measured plasmatic levels of vitamin D and AMH. This might be due to the possibility that plasmatic levels do not reflect what inherently occurs in tissues. In this regard, there was a study on women with infertility that showed a strong negative correlation of 25-hydroxyvitamin D levels in blood and follicular fluid [34].

### 3.2. Clinical Studies and Meta-Analyses

There have been several clinical observational and interventional studies showing significant relationships between vitamin D and ovarian reserve markers. In observational studies, a positive relationship between serum vitamin D and AMH levels was revealed in a cross-sectional study of 388 premenopausal women [35], and a negative correlation between serum vitamin D levels and urinary FSH levels was found in another study of 1430 premenopausal women [36], suggesting that lower vitamin D levels might be associated with lower ovarian reserve in late reproductive-age women (≥40 years) and earlier menopause. Dennis et al. also found that vitamin D might have a positive effect on AMH production in adults, and the seasonal changes in women’s AMH levels also correlates with those in vitamin D levels [37]. In recent interventional studies, improvements in ovarian reserve markers were reported with vitamin D supplementation, supporting a possible favorable effect of vitamin D on ovarian reserve markers [38,39]. However, there were also other studies that revealed no significant association between vitamin D and ovarian reserve markers, such as AMH and AFC [14,40,41]. Therefore, vitamin D still seems to have inconsistent evidence regarding its relation with ovarian reserves. Nonetheless, it is noteworthy that some recent studies, including meta-analyses, have reported promising results showing that vitamin D supplementation led to improvement in ovarian reserve metrics in a subgroup of normal or diminished ovarian reserves. There were three recent meta-analyses published between 2020 and 2022, two of which provided evidence that vitamin D supplements lead to improved ovarian reserve levels in a subgroup of non-PCOS women with normal ovulation or diminished ovarian reserves [42,43], and one found that lower AFC was associated with vitamin D insufficiency/deficiency in a subgroup analysis of Asians [44]. Moridi et al. highlighted that 18 cross-sectional studies had discrepant findings regarding an association between serum vitamin D and AMH levels, which is likely due to heterogeneity in study subjects and the complex nonlinear relationship between vitamin D and AMH in the systematic review. In contrast, they also demonstrated a cause–effect relationship between vitamin D supplements and AMH in the meta-analysis of six interventional studies, in which, interestingly, serum AMH was significantly increased in ovulatory non-PCOS women and decreased in PCOS patients after vitamin D supplementation [42]. Similarly, in other meta-analyses, vitamin D supplements led to an increase in AMH levels in non-PCOS women but no increase in AMH levels in PCOS patients [43].

The role of vitamin D in women with PCOS is even more noteworthy, considering that PCOS can be commonly accompanied by vitamin D deficiency in 67–85% of cases [45]. Vitamin D supplementation has been reported to reduce serum androgen and AMH levels and endometrial thickness [46] and to improve fertility indicators by increasing endometrial VDR expression and improving endometrial receptivity [47,48]. Zhao et al. showed that both implantation and clinical pregnancy occurrence were significantly higher in patients with normal vitamin D levels compared to patients with decreased vitamin D levels and that the number of high-quality embryos after vitamin D supplements was equivalent to the number of embryos in women with normal vitamin D levels [49].

**Table 1:** The association between vitamin D and ovarian reserve markers.

Study	Study Type/Design	Population	Summary
Kinuta 2000 [7]	Animal study	VDR null mutant mice (*n* = 3–10/group)	VDR null mutant mice: impaired folliulogenesis, uterine hypoplasia, decreased aromatase activity, and aromatase gene expression were observed.
Parikh 2010 [12]	Cell line (human)	Ovarian tissues from 26 women with infertility	Vitamin D stimulated steroidogenesis in human ovarian cells.
Wojtusik 2012 [27]	Cell line (human)	Follicles from hens (*n* = 3–7/group)	Vitamin D regulates AMH expression and may influence follicle selection in hens.
Merhi 2012 [35]	Cross-sectional study	388 premenopausal women with regular menses	Vitamin D was positively correlated with serum AMH levels in late reproductive women (≥40 years old).
Jukic 2015 [36]	Cross-sectional study	1430 premenopausal women, population-based	Vitamin D is inversely related to FSH.
Kim 2020 [14]	Prospective observational study	63 women with secondary amenorrhea	No correlation between vitamin D levels and OR markers, but vitamin D deficiency may be linked to PCOS patients.
Shapiro 2018 [41]	Retrospective cohort study	457 infertile women aged 21 to 50 years	Vitamin D was not associated with OR in infertile women with a high prevalence of diminished ORs.
Bacanakgil 2022 [38]	Prospective, nonrandomized, cross-sectional study	142 infertile women aged 18 to 41 years	Improvements in OR markers were reported with vitamin D supplementation.
Aramesh 2021 [39]	Before-and-after intervention study	51 infertile women aged 18 to 40 years	A statistically significant difference in serum AMH levels of participants after vitamin D supplementation.
Moridi 2020 [42]	Systematic review and meta-analysis	18 observational studies and 6 interventional studies	A total of 18 cross-sectional studies had discrepant findings, but there was a cause–effect relationship between vitamin D supplements and AMH in the meta-analysis of 6 interventional studies.
Yin 2022 [43]	Meta-analysis	51 self-control studies in women of reproductive age	After vitamin D treatment, the serum AMH increased, and it was obvious in non-PCOS patients.

VDR: vitamin D receptor; OR: ovarian reserve; AMH: anti-Müllerian hormone; FSH: follicle-stimulating hormone; PCOS: polycystic ovarian syndrome.

## 4. The Association of Vitamin D and Depression

Observational studies have suggested that women with low vitamin D levels are predisposed to PCOS, infertility, and endometriosis [50], as well as psychological disorders such as depression [51]. Women with reproductive diseases that affect fertility are known to have a higher prevalence of depression [52], so the relationship between vitamin D and depression in relation to fertility is also noteworthy. It is proposed that various mechanisms are involved in the pathogenesis of depression, including those affecting neuroendocrine, immunologic, neurotrophic, and metabolic systems [53]. Vitamin D is also thought to have a variety of functions, such as neuroimmunomodulation, regulation of neurotrophin, and neuroplasticity, in the brain [54] and to be involved in serotonin synthesis and maintenance of the circadian rhythm [55]. Moreover, VDR was found in neurons and glia in many regions of the brain (prefrontal cortex, substantia nigra, cingulate cortex, hippocampus, and hypothalamus) that may play a role in the pathophysiology of depression [8], and vitamin D is biologically suggested to not only be involved in the synthesis of neurotransmitters such as serotonin, dopamine, adrenalin, and noradrenalin through VDR but also to moderate the hypothalamic–pituitary–adrenal axis (HPA) and γ-aminobutyric acid A (GABA-A) receptor activity [56]. In this context, many studies have been conducted on the relationship between vitamin D and depression in the basic or clinical fields and in the meta-analyses as well. A summary of relevant studies is provided in Table 2.

### 4.1. Basic Studies in Cellular and Genetic Level

Although the pathophysiology of how vitamin D affects depression has not yet been fully elucidated, several main mechanisms have been reported. First, VDR and 1-α-hydroxylase (vitamin D activating enzyme) are widely distributed in the brain, especially in the hippocampus, which plays a key role in the mechanisms of depression [57]. Numerous studies conducted on in vitro hippocampal cells and in vivo adult rodents have shown that vitamin D deficiency alters the structure or function of the hippocampus during development [58]. Additionally, it has been demonstrated that vitamin D can regulate neurotrophins such as nerve growth factor (NGF), BDNF, and neurotrophin (NT)-3, which are essential for the survival and differentiation of neurons during development [59]. It has been reported that vitamin D may affect depression by increasing BDNF, which plays an important role in the survival, differentiation, and function of newborn neurons in the adult hippocampus [13,60]. Second, vitamin D could influence serotonin synthesis by alleviating tryptophan hydroxylase 2 (TPH2) and repressing tryptophan hydroxylase 1 (TPH1). It thus has an antidepressant effect by modulating the serotonergic system [61,62]. A strong body of evidence of the role of serotonin in the pathophysiology of depression has been built, and serotonin also acts on the hippocampus [63]. Vitamin D is known to be involved in depression by also affecting the levels of dopamine and noradrenalin [64]. Third, vitamin D is thought to control inflammation by reducing the expression of inflammatory cytokines, exhibiting a neuroprotective effect [65]. Grudet et al. have also shown that vitamin D insufficiency increased inflammatory markers such as IL-1β and IL-6 in depressive rats, although the underlying mechanisms are not clear yet [66]. In addition, vitamin D has been reported to be related to depression through mechanisms such as antioxidant effects in the CNS, regulation of expression of calcium homeostasis genes, regulation of mitochondrial protein expression, and regulation of demethylation, but additional studies and evidence seem to be necessary for further understanding [60].

### 4.2. Clinical Studies and Meta-Analyses on Cohort and Interventional Studies

There have been numerous observational studies on the relationship between serum vitamin D and depression and interventional studies on the effect of vitamin D supplements on depression. Several meta-analyses have also been conducted, which reflects the strong interest in the role of vitamin D. Observational studies have generally suggested an association between low serum vitamin D levels and depression. Vitamin D deficiency was related to depression-like symptoms, and subjects with anxiety or depression showed lower serum vitamin D levels [51,67]. Seasonal affective disorder was also suggested to be related to low vitamin D levels in northern latitudes with less sunlight exposure and in winter [68]. However, recent studies have failed to prove this relationship in female or elderly populations [69,70]. These discrepancies may result from not considering possible modulating factors such as subject characteristics and sociodemographic factors (sex, body mass index, diet, underlying diseases, drinking and smoking, etc.) and limitations of cross-sectional studies (biases caused by reverse causality: low vitamin D due to less outdoor activity/reduced nutrient intake, self-scored depression, unadjusted data, etc.). Interventional studies on the effect of vitamin D supplements in reducing depression have also provided inconsistent results. In this regard, several meta-analyses of randomized controlled trials (RCTs) of depressive patients were conducted, and some found a positive effect of vitamin D on depression severity [71,72], while others showed insignificant vitamin D efficacy for depressive symptoms [73,74]. However, interpretation of these results should also take into account that they also did not examine some factors that may modulate the efficacy of vitamin D in different settings. Also, considering there is reciprocity between vitamin D and epigenetic mechanisms [75], there may be a complex nonlinear relationship between vitamin D and reproduction or depression due to epigenetic changes caused by vitamin D supplementation. Recently, Musazadeh et al. conducted an umbrella meta-analysis on ten meta-analyses of interventional RCTs and four meta-analyses of cohort observational studies [76]. This umbrella meta-analysis demonstrated a significant reduction in depressive symptoms in patients with vitamin D supplements and increased odds of depression in patients with low serum vitamin D levels. In another recent meta-analysis of 18 RCTs, vitamin D supplements were effective in depressed patients in heterogeneous data [77]. In these two recent meta-analyses, there were subgroup analyses of several factors that might modulate vitamin D efficacy, and no differences in the efficacy of vitamin D supplementation according to gender were observed, suggesting that vitamin D supplementation is beneficial to both men and women [76,77]. To date, there is growing promise that low vitamin D levels are related to the risk of depression and that vitamin D supplementation may be effective in treating depression. Table 3 summarizes the results of the review on the relationship between vitamin D, ovarian reserves, and depression, as discussed above.

**Table 2:** The association between vitamin D and depression.

Study	Study Type/Design	Population	Summary
Koshkina 2019 [13]	Animal study	Ovariectomized rats (*n* = 7/group)	Vitamin D could improve the depression profile in ovariectomized rats by regulating BDNF.
Neis 2022 [62]	Animal study	Female Swiss mice (*n* = 7–8/group)	Vitamin D has an antidepressant-like effect by modulating serotonin.
Gessa 2021 [67]	Prospective cohort study	3365 participants aged 50 and over (English Longitudinal Study of Aging)	Subjects with lower vitamin D are more likely to be depressed than those with high levels of vitamin D.
Bičíková 2015 [51]	Cross-sectional study	64 men and 86 women with depressive/anxiety disorders and healthy controls	Significantly lower vitamin D levels were found in patients with depression as well as in age-matched patients with anxiety disorders.
Rhee 2020 [69]	Cross-sectional study	820 men and 916 women, aged 19 to 76	Serum vitamin D levels were inversely associated with cognitive/affective depressive symptoms only in men.
Mikola 2022 [71]	Systematic review and meta-analysis	41 RCTs (*n* = 52,235)	Vitamin D supplements have the effect of reducing depression symptoms.
Tomé 2021 [73]	Systematic review and meta-analysis	10 RCTs (*n* = 1393)	Vitamin D did not have a significant therapeutic effect on depression.
Musaadeh 2023 [76]	Umbrella meta-analysis	10 meta-analyses of interventional RCT and 4 meta-analyses of cohort observational studies	Vitamin D supplements significantly reduced depressive symptoms, and participants with lower vitamin D levels had increased risk of depression.

BDNF: brain-derived neurotrophic factor; RCTs: randomized controlled trials.

## 5. Is There Any Potential Role of Vitamin D in Depression Related to Female Reproductive Hormones?

One of the causes of excessive depression in women compared to men is that depression can commonly occur due to changes in ovarian hormones, such as the menstrual cycle, pregnancy, and menopause. These clinically manifest as premenstrual depression (PMS)/premenstrual dysphoric disorder (PMDD), postpartum depression, and menopausal depression, which Nappi et al. referred to as ‘reproductive depression’ [21]. The marked hormonal fluctuations exert a profound effect on brain areas relevant for mood, memory, and behavioral/cognitive responses by influencing neurotransmission, neuromodulation, synaptic plasticity, and neurodegeneration [78]. Estrogen affects multiple neural pathways, including serotonergic, dopaminergic, noradrenergic, cholinergic, GABAergic, etc., and progesterone and its neuroactive metabolites are also active at the GABA-A receptor to modulate mood changes [79,80]. More specifically, estrogen is known to regulate BDNF, neurotransmitter-synthesizing enzymes, neurotransmitter-metabolizing enzymes, and their receptors by acting on Estrogen Receptor α (ERα) and ERβ distributed in various parts of the brain, including depression-related hypothalamus, hippocampus, and serotonin neurons of dorsal raphe [81,82]. In this way, estrogen exerts neuroprotective activity by positively affecting serotonergic raphe neurons, noradrenergic networks, and the dopamine system [83,84,85,86]. Estrogen is also known to strongly regulate adrenal, thyroid, and circadian rhythm functions related to depression [87,88]. In addition to depression related to physiological sex hormone fluctuations, there have been many reports that some female reproductive diseases (PCOS, endometriosis, POI, etc.) with sex hormone abnormalities are also related to depression [18,89,90]. Dybcik et al. showed a two-and-a-half times higher risk of depression in PCOS patients compared to healthy women in the recent meta-analysis of 4002 patients from 19 studies [89], and Allshouse et al. reported that POI patients had an increased risk of depression [18].

**Figure 2:**
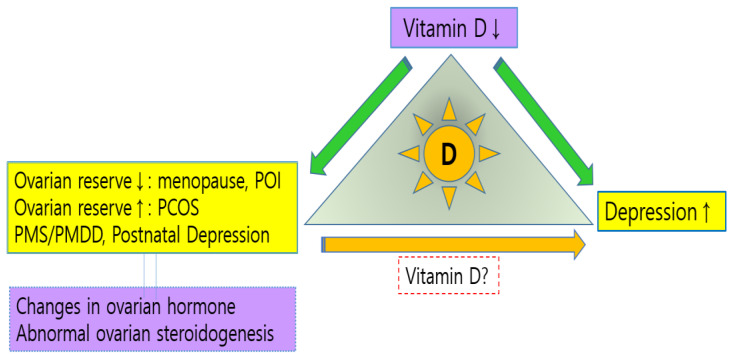
Schematic diagram showing the relationship of vitamin D with both ovarian hormone changes/abnormalities and depression.

Since these sex hormonal changes in female physiologic and pathologic conditions would be ultimately related to a decrease or increase in ovarian reserves, vitamin D, which is thought to be related to both ovarian reserve and depression, could also be assumed to be another important part of the mechanism of depression related to female reproductive hormones (Figure 2). Moreover, as seen earlier, vitamin D and ovarian hormones have similarities in the mechanisms involved in depression, such as the distribution of their receptors and neuroprotective effects in the brain. Indeed, Kolhe et al. have suggested a possibility of common associations between depression and PCOS and a potential role of vitamin D in the depression of PCOS patients [91]. However, there is still a lack of evidence regarding a causal relationship between vitamin D and depression in these patients. In our previous study, we showed that low levels of vitamin D and AMH were associated with depressive severity in patients with secondary amenorrhea, but the cross-sectional study could not suggest a causal relationship with depression [22]. Additionally, research results on the effects of vitamin D supplementation on depression in postmenopausal or PCOS women are still inconsistent [23,92,93,94]. It is anticipated that further studies in standardized settings, controlling for the various factors involved in depression, will shed light on the role of vitamin D in depression related to female reproductive hormones.

## 6. Role of Microbiota in Linking Vitamin D and Female Reproduction/Depression

As seen above, many studies have suggested that vitamin D is related to ovarian reserve markers and depression, but some have shown inconsistent results. Here, another important factor to consider in the relationship between vitamin D and female reproduction or depression is the role of microbiota, which has recently received much interest. In recent years, many clinicians and scientists have focused on the role of the microbiome in the pathogenesis and prevention of various diseases, and many studies have been conducted. Gut microbiomes are known to be responsible for estrogen metabolism, as microbe-secreted β-glucuronidase converts conjugated estrogen into deconjugated estrogen, as shown in [95]. The binding of deconjugated estrogens to ERs ultimately affects reproductive health and neural development [96]. Furthermore, some microbiomes increase inflammatory mediators, which upregulate the expression of enzymes involved in ovarian steroidogenesis and interact with estrogen; thus, they can induce gynecologic disorders [97,98]. Indeed, a strong relationship between the microbiome and estrogen-related diseases or states (endometrial cancer, endometriosis, uterine fibroids, PCOS, and postmenopausal syndrome) has been demonstrated [99,100,101,102,103,104]. In addition, many reports indicate that the gut microbiota affects mood and brain health by regulating immune, neuroendocrine, and neural pathways, which are components of the brain–gut–microbiota axis [105,106], and altered gut microbiota is associated with depression and anxiety [107,108].

Diet and nutrition are important factors in maintaining microbiome homeostasis in the body, and vitamin D has been noted as a nutrient essential for maintaining gut microbiome homeostasis [109]. Therefore, vitamin D deficiency can cause dysbiosis of the gut microbiota. Thus, studies have recently reported that vitamin D as a dietary intervention is effective in preventing or treating female reproductive diseases and depression through the estrogen–gut microbiome axis and brain–gut microbiome axis [110,111]. In the context of these findings, when evaluating the relationship between vitamin D and female reproduction/depression, it is important to remember that the state of the body’s microbiome is an important factor that can link their relationship.

## 7. Recommendations and Summary

Several major health authorities have developed recommendations for vitamin D supplements and guidance about optimal serum 25-hydroxyvitamin D [25(OH)D] concentrations. The U.S. Institutes of Medicine (IOM) recommended a target 25(OH)D concentration of 20 ngmL (50 nmol/L) focused on bone health and recommended 400 IU/day for infants; 600 IU/day for children, adolescents, and adults; and 800 IU/day for adults aged over 70 years to maintain a desirable 25(OH)D concentration [112]. However, the guidelines from most other societies focused on the pleiotropic effects of vitamin D recommended a target 25(OH)D concentration of 30 ng/mL (75 nmol/L). The Endocrine Society in the U.S. recommended 400–1000 IU/day for infants, 600–1000 IU/day for children over 1 year, and 1500–2000 IU/day for all adults. It was also recommended for obese people (BMI > 30 kg/m^2^) to take a three times greater dose than the recommended dose for subjects with a normal body weight [113]. The optimal vitamin D level and recommended vitamin D intake dose for PCOS women are still controversial, and there have been several reports on vitamin D supplementation at various concentrations, but some have suggested that lower concentrations of vitamin D (400–800 IU/day) may be beneficial [48].

The significant association of vitamin D with ovarian reserve markers and depression has been revealed in numerous basic or clinical studies, but some observational and interventional clinical studies have shown inconsistent results. These results may be due to the heterogeneity of these studies in study subjects, study design, intervention, ovarian reserve marker or depression measurement methods, vitamin D status of study subjects, and modulating factors considered. Nevertheless, recent meta-analyses of interventional studies have provided promising results showing that vitamin D supplementation significantly improves AMH, especially in a subgroup of women with normal or diminished ovarian reserves, and decreases depressive symptoms and risk. The demonstration of an association of vitamin D with both ovarian reserves and depression could suggest that vitamin D may be another important key in explaining female reproductive depression, but more research is expected on this topic. Larger-scale studies in standardized settings will be needed in order to gain further insight into the role of vitamin D in female reproduction and depression.

## Figures and Tables

**Table 3:** Summary of review on the relationships of vitamin D with ovarian reserves and depression.

		Vitamin D and OR Markers	Vitamin D and Depression
Cellular/Genetic studies		Associated (positive)	Associated (negative)
Serum levels	Observational studies	Associated with low OR/late reproductive age; some inconsistent	Generally associated; discrepancies in women, elderly populations
	Interventional studies	Beneficial effects in normal or diminished OR; some inconsistent	Inconsistent
Meta-analyses		Beneficial effects (AMH ↑ in non-PCOS, AMH ↓ or ↔ in PCOS)	Beneficial effects

OR: ovarian reserve; AMH: anti-Müllerian hormone; PCOS: polycystic ovarian syndrome; ↑: increase; ↓: decrease; ↔: no significant change.
